# The prevalence of secondhand smoke exposure and related factors among schoolchildren in Northeast Thailand

**DOI:** 10.12688/f1000research.26039.1

**Published:** 2020-09-21

**Authors:** Nirun Intarut, Piyalak Pukdeesamai

**Affiliations:** 1 Health System Science Unit, Faculty of Medicine, Masarakham University, Mueng, Mahasarakham, 44000, Thailand; 2 Phanomphrai Hospital, Phanom Phrai. Roi Et, 45140, Thailand

**Keywords:** schoolchildren, adolescent, secondhand smoke exposure, depression, number of smokers

## Abstract

**Background:** The prevalence of tobacco consumption in Thailand has gradually declined; however, the prevalence of exposure to secondhand smoke (SHS) is still high. The objective of this study is to estimate the prevalence of SHS exposure and examine the association between exposure to SHS and depressive symptoms among schoolchildren, and test for moderation by the number of smokers in household.

**Methods:** We conducted a cross-sectional study of 1105 schoolchildren. Socioeconomics factors, depressive symptoms and exposure to SHS variables were collected. We used the chi-square test for testing the factors associated to SHS exposure. In addition, we used the Mantel Haenszel test for testing interaction effect of depression to SHS exposure by the number of smokers in home. Multiple logistic regression was used to test the factors related to SHS exposure adjusted for confounders.

**Results:** The prevalence of exposure to SHS was 58.2% (95%CI: 55.2, 61.1). The schoolchildren with abnormal depression status were 1.8 times more likely to have been exposed to SHS (95%CI: 1.3, 2.5). In addition, the number of smokers in the home did not modify the association between exposure to SHS and depressive symptoms (P: 0.964).

**Conclusions:** An association between exposure to SHS and depressive in schoolchildren was observed, but this relationship was not affected by the number of smokers in children’s homes.

## Introduction

 In Thailand, the prevalence of tobacco consumption among people aged 15 years old or above has decreased from 32.0% in 1991 to 19.1% in 2017; however, the prevalence of exposure to secondhand smoke (SHS) in the home remains quite high, 32.7% in 2017 ^1^. Exposure to SHS has been linked to several diseases including cancer and heart disease, especially in children and non-smokers ^2^. Many strategies have been implemented to reduce non-smokers’ exposure to SHS, including legislation to prevent smoking in public places and health promotion ^3^. Laws have been passed to ban smoking on public transport, in airports, in restaurants, and in schools; however, this legislation does not cover private places such as the home.

 Home is a place where family members in a range of activities, and is a common location for exposure to SHS ^4– 6^. Most studies to date show that exposure to SHS is linked to depression, including SHS exposure occurring in the home ^7– 11^. Just one study found no such connection ^12^. Limited data is presently available on the association between SHS exposure and depression in schoolchildren. We therefore conducted this study to investigate whether or not there is an association between SHS exposure and depressive symptoms in this population, and whether the number of smokers in children’s households affects this association.

## Methods

### Study design

This cross-sectional survey was conducted in nine schools located in northeast Thailand from August in 2018 to March in 2019.

### Sample size calculation

We calculated the sample size for this survey as the following formula: $n=(z_{\alpha /2}^{2}\times p\times q)/d^{2}$ in which α is 0.05; p is 0.232 that was reported from Leung L *et al.* ^13^, q=1-0.232=0.768; d=0.035. In addition, we multiplied the total sample size with design effect 1.5. So, in total at least 900 participants were needed.

### Data collection

 Schools were selected by simple random sampling from the list of schools provided by the Office of the Basic Education Commission (OBEC). We contacted nine schools to describe our study and invite them to participate in the study. In each school, students in grades 6 to 8 (13 to 15 years old), were asked to participate in the study. The rationale for using these grades was that students of this age group still mostly stay within their homes outside of school and therefore would be more likely to be exposed to SHS if a smoker was present in the household. The total number of students in grade 7 (Mattayomsuksa 1), grade 8 (Mattayomsuksa 2), and grade 9 (Mattayomsuksa 3) from the selected school was 2278. All were asked to complete the questionnaire by themselves following instructions from a research assistant. Students that were part of the same household as an existing participant or had any psychiatric disorders as identified by their teacher were excluded. After filling in the self-report questionnaire, students were asked to deliver questionnaires to their guardians to collect information on household variables. A guardian was defined as mother, father, or relation. They were asked to return these questionnaires to a teacher within 7 days. The questionnaire for student and parents are available as extended data ^14^. A total of 1103 families completed the questionnaires, equating to a response rate of 48.4% (1103/2278). We excluded 4 cases because there was not reporting of SHS exposure, so a total of 1099 participants were analyzed. Participants provided written informed consent form to participate in this study. A consent form was provided to the children who then took them home for their parents/guardians to review. This included a consent form for the parent/guardian and assent form for the child to complete. The child then returned these to school.

### Measurement

 Depressive symptoms were measured using the Center for Epidemiological Studies-Depression Scale (CESD) questionnaire. This questionnaire comprises of 20 questions and has a Cronbach's alpha of 0.86, and was translated into the Thai language ^15^. For each question, schoolchildren could respond never (0-score), rarely (1-score), often (2-score), or all the time (3-score). The scores for each question were summed, with totals ranging from 0 to 60. If the total scores of the CESD were more than 22, the schoolchildren were identified as having depressive symptoms ^16^.

For exposure to SHS, the schoolchildren were asked “In the past 7 days, have you inhaled any tobacco smoke?” The available answers were “inhaled on 1–3 days”, “inhaled on 4–6 days”, “inhaled every day”, or “No”. Schoolchildren who reported “no” were classified as not having been exposed to SHS, and the others were classified as having been exposed to SHS. We also collected information on variables such as the number of years of school attended, household’s income per month, number of smokers in the home, school grade (grade 7, grade 8, and grade 9), perceived danger of exposure to SHS and third hand smoke, self-confidence in avoidance of SHS (scores ranged 0-20), SHS attitude (scores ranged 0-10), SHS knowledge (scores ranged 0-10), and place of exposure to SHS.

### Data analysis

 The association between variables and exposure to SHS were examined using Chi-square tests or Fisher’s exact tests as appropriate. We also tested the effect of the number of smokers per household on the association between depressive symptom and exposure to SHS by using the Mantel-Haenszel Test. For multivariable analysis, we used multiple logistic regression for adjusting potential confounders. All data analysis was performed using R version 3.6.0 ^17^.

## Ethics

The Mahasarakham University Research Ethics Board provided ethical approval for this study (Approval number:115/2018).

## Results

A total of 1099 subjects were included in the analysis. The prevalence of exposure to SHS was 58.2% (95%CI: 55.2, 61.1). In this study, 92.9% of guardians had attended 1–6 years of school, 85.2% of participants had a household income less than 10000 Thai Baht (THB; equivalent to 218.37 USD at time of publication), and 20.8% of households had more than one person who was a smoker. Most students were in 7 ^th^ grade (39.8%), and 63.4% were female. Awareness of the harm caused by SHS exposure and third hand smoke exposure was 35.1% and 57.2%, respectively. The mean scores of self-confidence in avoidance of SHS was 12.7 (SD: 5.5). The mean scores of attitude and knowledge of secondhand smoke exposure were 27.5 (SD: 3.1) and 4.5 (SD: 1.3), respectively. The characteristics of the participants are shown in Table 1 and full details for each participant available as underlying data ^14^.

**Table 1.** Characteristics and univariate analysis of participants by exposure to secondhand smoke.

	Total n=1099	No eSHS n=456	eSHS n=643	P value
Household factors				
Relation to participant Mothe Father Relative	179 (16.3) 454 (41.3) 466 (42.4)	75 (16.4) 186 (40.8) 195 (42.8)	104 (16.2) 268 (41.7) 271 (42.1)	0.957
The number of years attended in school 0 1-6 ≥ 7	15 (1.4) 1024 (93.2) 60 (5.5)	6 (1.3) 421 (92.3) 29 (6.4)	9 (1.4) 603 (93.8) 31 (4.8)	0.54
Household income per month (Thai baht) <10,000 ≥ 10,000	941 (85.6) 158 (14.4)	385 (84.4) 71 (15.6)	556 (86.5) 87 (13.5)	0.342
Number of smokers in house (persons) None 1 ≥2	399 (36.3) 472 (42.9) 228 (20.7)	216 (47.4) 147 (32.2) 93 (20.4)	183 (28.5) 325 (50.5) 135 (21.0)	< 0.001
Schoolchildren factors				
School grade placement Grade 7 Grade 8 Grade 9	437 (39.8) 322 (29.3) 340 (30.9)	181 (39.7) 127 (27.9) 148 (32.5)	256 (39.8) 195 (30.3) 192 (29.9)	0.565
Gender Male Female	403 (36.7) 696 (63.3)	190 (41.7) 266 (58.3)	213 (33.1) 430 (66.9)	0.004
Perception about the harm of eSHS Strong disagree Disagree Agree Strongly agree	69 (6.3) 62 (5.7) 385 (35.1) 581 (53)	29 (6.4) 23 (5.1) 166 (36.6) 236 (52.0)	40 (6.2) 39 (6.1) 219 (34.1) 345 (53.7)	0.778
Perception about the harm of exposure to third hand smoke Strong disagree Disagree Agree Strongly agree	58 (5.3) 108 (9.8) 628 (57.2) 304 (27.7)	29 (6.4) 34 (7.5) 255 (56) 137 (30.1)	29 (4.5) 74 (11.5) 373 (58.0) 167 (26.0)	0.046
Self-confidence in avoidance of eSHS score Mean (SD) Median (Q1,Q3)	12.7 (5.5) 13 (10, 17)	13.3 (5.7) 14 (10, 18)	12.3 (5.2) 12 (9,16)	< 0.001
SHS attitude Mean (SD) Median (Q1,Q3)	27.5 (3.1) 28 (25, 30)	27.5 (3.1) 28 (25, 30)	27.5 (3.1) 28 (26, 30)	0.636
SHS knowledge Mean (SD) Median (Q1,Q3)	4.5 (1.3) 5 (4, 5)	4.5 (1.4) 5 (4, 6)	4.5 (1.3) 5 (4, 5)	0.513
Place of exposure Home Friend’s home School Temple Market Public transport Other Multiple exposure No exposure 1 place ≥ 2 places	409 (37.2) 108 (9.8) 103 (9.4) 12 (1.1) 88 (8.0) 21 (1.9) 47 (4.3) 456 (41.5) 518 (47.1) 125 (11.4)	0 (0) 0 (0) 0 (0) 0 (0) 0 (0) 0 (0) 0 (0) 456 (100) 0 (0) 0 (0)	409 (63.6) 108 (16.8) 103 (16.0) 12 (1.9) 88 (13.7) 21 (3.3) 47 (7.3) 0 (0) 518 (80.6) 125 (19.4)	
Center for Epidemiological Studies Depression (CESD) Normal Abnormal	878 (79.9) 221 (20.1)	390 (85.5) 66 (14.5)	488 (75.9) 155 (24.1)	<0.001

eSHS = Exposure to secondhand smoke

 In Table 1, information is presented on the association between participant risk factors and SHS exposure. The results show there was a statistically significant association between SHS exposure and the number of smokers per household (P value: < 0.001), gender (P value: 0.004), awareness of the harm caused by third hand smoke exposure (P value: 0.046), and self-confidence in avoidance of SHS exposure (P value < 0.001). A statistically significant association was not detected between SHS exposure and relation of the parent/guardian to the school child (P value: 0.957), the number of years attended in school (P value: 0.540), household income per month (P value: 0.342), school grade placement (P value: 0.565), perception about the harm of eSHS (P value:0.778), SHS attitude (P value:0.636), and SHS knowledge (P value:0.513). In Table 2, we present results from the multiple logistic regression model to control for confounder factors. Our analysis shows that schoolchildren with an abnormal CESD state are 1.8 times more likely to have been exposed to SHS (95%CI: 1.3, 2.5; P value <0.001) than those with a normal CESD state, with adjustment made for the number of years of school attended, household income per month, perception about the harm caused by SHS exposure, perception about the harm caused by third hand smoke exposure, SHS attitude, SHS knowledge, and self-confidence in avoidance of SHS exposure.

**Table 2.** Effect of the number of smokers per household on the association between exposure to secondhand smoke and depression.

Number of smokers in house	No depression	Depression	OR (95%CI)	P value
	N with/without eSHS	N with/without eSHS		
0	145/188	38/28	1.8 (1.0, 3.1)	0.043
1	241/124	84/23	1.9 (1.1, 3.3)	0.017
≥2	102/78	33/15	1.7 (0.8, 3.6)	0.140

Homogeneity test (Mantel-Haenszel Testing), P value = 0.964, eSHS = Exposure to secondhand smoke

We also investigated the effect of the number of smokers per household. In households with no smokers, schoolchildren who reported depressive symptoms were 1.8 times (95%CI: 1.0, 3.1) more likely to exposed to SHS than those who were not. In households with one smoker, schoolchildren who had depressive symptoms were 1.9 times (95%CI: 1.1, 3.3) more likely to exposed to SHS. Lastly, in households with two or more smokers, schoolchildren who reported depressive symptoms were 1.7 times (95%CI: 0.8, 3.6) more likely to be exposed to SHS than those who reported no depressive symptoms ( Table 3). The results of the Mantel-Haenszel test show there was no statistically significant difference between the number of smokers per household and the association between SHS exposure and depression symptoms.

**Table 3.** Association between exposure to secondhand smoke and depression symptoms.

	n/N	Crude OR (95%CI)	Adjusted OR (95%CI)
Depression symptoms Normal Abnormal	55.6% (488/878) 70.1% (155/221)	1 1.9 (1.4, 2.6)	1 1.8 (1.3, 2.5)
Number of smokers in house 0 1 ≥2	45.9% (183/399) 68.9% (325/472) 59.2% (135/228)	1 2.5 (1.9, 3.3) 1.8 (1.3,258)	1 2.5 (1.9, 3.3) 1.7 (1.2, 2.4)

Adjusted for the number of years attended in schools, household income per month, perception about the harm of SHS exposure, perception about the harm of third hand smoke exposure, SHS attitude, SHS knowledge, and self-confidence in avoiding SHS exposure scores.

## Discussion

 This study shows there is a high prevalence of SHS exposure in schoolchildren in northeast Thailand, and a statistically significant association between SHS exposure and depressive symptoms in these children. The number of smokers per household did not affect this association.

 The prevalence of SHS exposure in this study was 58.2%, a higher rate than a comparable study in the US where exposure was 48% ^18^, and a study in lower-middle-income countries where exposure was 55.9% ^19^. The prevalence of tobacco use in Thailand has declined gradually, but the prevalence of SHS exposure is still quite high ^1^. This may be due to non-smokers not being aware of the harms of SHS exposure or to smokers continuing to smoke in locations near non-smokers.

 When a univariate analysis was performed to test the association between different factors and SHS exposure, and SHS exposure and depressive symptoms, several statistically significant associations were identified. After adjusting for potential confounding factors, we also showed that schoolchildren exposed to SHS tend to have more depressive symptoms than unexposed children. This finding is similar to several previous studies ^7, 20– 22^, except one conducted in the Netherlands ^12^. Possible reasons for the disparity include differences in sample population and depressive symptom measurement. Previous studies that detected an association between SHS exposure and depressive symptoms used a predominantly adolescent population ^7, 20– 22^, while the study that did not used an adult population. It may be that children and adolescents who are exposed to SHS are not in a position to move away from the smoker, and depressive symptoms are stimulated. Adults, by comparison, have greater autonomy and can more readily avoid SHS exposure.

Our study also investigated whether the number of smokers in a household affects the association between SHS exposure and depression. No interactive effect was detected. The association between SHS exposure and depression might be of equal magnitude regardless of the number of household smokers. Very few studies consider the interaction between known risk factors and the association between SHS exposure and depression. Our assumption was that, if there are more smokers in a household, there would be a greater chance of SHS exposure and depression. 

## Strengths and limitations

This study has two major strengths. It is the first study to examine how the number of smokers per household affects the association between SHS exposure and depressive symptoms. Also, the sample size was large, so the findings should be more accurate than smaller-sized studies. Set against these two strengths are the following three limitations. Firstly, the schoolchildren were selected from northeast Thailand, and may not be representative of schoolchildren nationally. Secondly, a cross-sectional study design was used, so we cannot conclude there is a causal relationship between SHS exposure and depressive symptoms. Finally, the survey relied on self-reports, so there may be recall bias with regard to history of exposure.

## Conclusion

 In this study, we observed the association between SHS exposure and depressive symptoms. However, the number of smokers per household did not affect this association.

## Data availability

### Underlying data

Harvard Dataverse: Secondhand smoke: https://doi.org/10.7910/DVN/I7KCVH ^14^

This project contains the following underlying data:

-    datashsfandcesd1000research.tab (data secondhand smoke and cesd – not that Grade 1, 2 and 3 correspond with grade 7 (Mattayomsuksa 1), grade 8 (Mattayomsuksa 2), and grade 9 (Mattayomsuksa 3))

### Extended data

Harvard Dataverse: Secondhand smoke: https://doi.org/10.7910/DVN/I7KCVH ^14^

This project contains the following extended data:

-    parent_questionaire_eng.pdf (parent questionnaire English)

-    parent_questionaire_thai.pdf (parent questionnaire Thai)

-    student_questionaire_eng.pdf (student questionnaire English)

-    student_questionaire_thai.pdf (student questionnaire Thai)

Data are available under the terms of the Creative Commons Zero "No rights reserved" data waiver (CC0 1.0 Public domain dedication).
