# Supplementary material for: The prevalence of secondhand smoke exposure and related factors among schoolchildren in Northeast Thailand
Source: F1000Res. 2020 Sep 21;9:1158. [Version 1] doi: 10.12688/f1000research.26039.1 (PMC7590888; doi:10.12688/f1000research.26039.1)
Supplement: Peer review history

# Peer review: Reviewer response for version 1

**DOI:** 10.5256/f1000research.28736.r71751

**Published:** 2020-10-26

**Authors:** Roongnapa Khampang

**Affiliations:** 1 Health Intervention and Technology Assessment Program, Nonthaburi, Thailand

**Introduction:**

- Could you please elaborate more on the rationale of the study and the significance of the study to public health? Why did you choose to study the association between SHS and depression instead of other diseases? Is there a lack of evidence in Thailand? Could you also provide a bit more detail on the psychological or biological mechanisms of SHS and depression so that the readers understand why depression is important here and how could SHS associate with depressive symptoms?
- In the method, you stated that you selected nine schools from northeast Thailand, please also provide detail in the introduction why you are interested in this area and how your findings would benefit the responsible organizations?

**Methods:**

- You stated that 9 schools were randomly selected from a list of schools but did not show the distribution of the schools among provinces in northeast Thailand. Were there any schools located in the same province? What were the sizes of the school included in the study? How many schools were located in urban areas and how many were in rural areas? This detail may help readers to understand and justify the representativeness of the study samples and the generalizability of the findings especially the prevalence of SHS.
- There is no detail on how the students (unit of analysis) were selected. Were all eligible students recruited?

**Results:**

- There are some typos in the manuscript such as in Table 1, mother, strongly disagree.
- There is no information on current smoking status of the students who were interviewed. Many studies found a positive association between smoking and depression and anxiety. Would this missing information influence the main findings?

**Discussion:**

- You may compare the characteristics of your study samples (both schools and students) with the figures of northeast Thailand to clarify about similarity of your samples and the population of northeast Thailand.
- How would the smoking status of the students influence your findings?
- It would be nice if you could develop policy recommendations drawing from your findings.

Is the work clearly and accurately presented and does it cite the current literature?

Yes

If applicable, is the statistical analysis and its interpretation appropriate?

Yes

Are all the source data underlying the results available to ensure full reproducibility?

Partly

Is the study design appropriate and is the work technically sound?

Partly

Are the conclusions drawn adequately supported by the results?

Yes

Are sufficient details of methods and analysis provided to allow replication by others?

Partly

Reviewer Expertise:

Epidemiology, health policy or program evaluation

I confirm that I have read this submission and believe that I have an appropriate level of expertise to confirm that it is of an acceptable scientific standard, however I have significant reservations, as outlined above.

# Peer review: Reviewer response for version 1

**DOI:** 10.5256/f1000research.28736.r71747

**Published:** 2020-10-08

**Authors:** Hendra Kurniawan

**Affiliations:** 1 Public Health and Community Medicine Department, Syiah Kuala University, Banda Aceh, Indonesia

- In the background: please clarify clearly which province in Thailand that covers the northeast Thailand area?
- If you used the CESD questionnaire, did you translate it to Thai language and validate it before or did you already have the valid Thai version?
- The category of income; household income less than 10000 Thai Baht, is mean enough or which category? Please state in your discussion part.

Is the work clearly and accurately presented and does it cite the current literature?

Yes

If applicable, is the statistical analysis and its interpretation appropriate?

Yes

Are all the source data underlying the results available to ensure full reproducibility?

Yes

Is the study design appropriate and is the work technically sound?

Yes

Are the conclusions drawn adequately supported by the results?

Yes

Are sufficient details of methods and analysis provided to allow replication by others?

Partly

Reviewer Expertise:

public health, pulmonology, health social study

I confirm that I have read this submission and believe that I have an appropriate level of expertise to confirm that it is of an acceptable scientific standard.

## References

[1] : Guidelines for developing, translating, and validating a questionnaire in perioperative and pain medicine. *Saudi J Anaesth*.2017;11(Suppl 1) : 10.4103/sja.SJA_203_17 S80-S89 10.4103/sja.SJA_203_17 28616007PMC5463570

# Peer review: Reviewer response for version 1

**DOI:** 10.5256/f1000research.28736.r71749

**Published:** 2020-10-06

**Authors:** Kyaw Ko Ko Htet

**Affiliations:** 1 Department of Medical Research (Pyin Oo Lwin Branch), Ministry of Health and Sports, Pyin Oo Lwin, Myanmar

I have some comments:

 1) Introduction:

- Why do the investigators want to investigate the association between the secondhand smoke exposure and depression among the children? It would be better to explain the magnitude of depression among children in the introduction part. 

2) Methods:

- What is the meaning of p is 0.232?
- How can response rate 48.4% affect the study result? Is it a study limitation?

3) Measurement:

- For information about number of smokers in household, who completed this information? It is not clear which information was completed by children and household members. 
- In some countries, grade 8 & 9 students have experience on smoking, no information was found in this study. Can it affect the study result?

4) Results:

- Table 2: It may need to explain how schoolchildren were exposed to secondhand smoke although there was no smoker in the house.
- The authors may need to explain the definition of second-hand smoke and third-hand smoke. In the result on perception about the harm of exposure to thirdhand smoke, is it third- or second-hand smoke?

Is the work clearly and accurately presented and does it cite the current literature?

Yes

If applicable, is the statistical analysis and its interpretation appropriate?

Yes

Are all the source data underlying the results available to ensure full reproducibility?

Yes

Is the study design appropriate and is the work technically sound?

Yes

Are the conclusions drawn adequately supported by the results?

Yes

Are sufficient details of methods and analysis provided to allow replication by others?

Yes

Reviewer Expertise:

Public health, NCD, TB

I confirm that I have read this submission and believe that I have an appropriate level of expertise to confirm that it is of an acceptable scientific standard, however I have significant reservations, as outlined above.

# Peer review: Reviewer response for version 1

**DOI:** 10.5256/f1000research.28736.r71748

**Published:** 2020-10-06

**Authors:** Pongdech Sarakarn

**Affiliations:** 1 Epidemiology and Biostatistics Department, Faculty of Public Health, Khon Kaen University, Khon Kaen, Thailand; 2 ASEAN Cancer Epidemiology and Prevention Research Group (ACEP), Faculty of Public Health, Khon Kaen University, Khon Kaen, Thailand

I have some comments and issues for adding and revising as follows;

1. In introduction, I don't find the literature regarding depression in schoolchildren, could you please add for completion?
2. In data collection, "in grades 6 to 8 " or "in grade 7 to 9", please clarify for this words.
3. Please clarify regarding the number of smokers in house (persons) and place of exposure in home (Table 1). I think both of these variables should be related (e.g. the number of place of exposure is 409, but the number of smokers in house (persons) (including 1,>=2) is 460).
4. In the results (page 5), please check the position of table; may mistake between "table2" and "table3".
5. In practice, I think not only the number of smokers in house (persons) can affect the exposure of smoking, but also the quantity of smoking (per persons) can relate as well. Therefore, this point should be added in the discussion or limitation parts of this study.
6. Finally, this study uses depression of schoolchildren for analyzing with the prevalence of secondhand smoke exposure, in my opinion "the depression of schoolchildren" should be added in the title name, if possible.

These preliminary results can be important for explanation of the effects of smoking. I think this paper can be useful, especially the schoolchildren group.

Is the work clearly and accurately presented and does it cite the current literature?

Yes

If applicable, is the statistical analysis and its interpretation appropriate?

Yes

Are all the source data underlying the results available to ensure full reproducibility?

Yes

Is the study design appropriate and is the work technically sound?

Yes

Are the conclusions drawn adequately supported by the results?

Partly

Are sufficient details of methods and analysis provided to allow replication by others?

Yes

Reviewer Expertise:

Biostatistics.

I confirm that I have read this submission and believe that I have an appropriate level of expertise to confirm that it is of an acceptable scientific standard, however I have significant reservations, as outlined above.
